# Supplementary material for: Non-Volatile Transistor Memory with a Polypeptide Dielectric
Source: Molecules. 2020 Jan 23;25(3):499. doi: 10.3390/molecules25030499 (PMC7036792; doi:10.3390/molecules25030499)
Supplement: Supplementary file 1 [file molecules-25-00499-s001.pdf]

Non-Volatile Transistor Memory with a Polypeptide Dielectric

Lijuan Liang [1,*], Wenjuan He [1], Rong Cao [1], Xianfu Wei [1], Sei Uemura [2], Toshihide Kamata [2], Kazuki Nakamura [3], Changshuai Ding [4], Xuying Liu [4] and Norihisa Kobayashi [3,*]

[1] Beijing Institute of Graphic Communication, Beijing 102600, China; h18736293925@hotmail.com (W.H.); c17812061939@outlook.com (R.C.); weixianfu@bigc.edu.cn (X.W.)

[2] Flexible Electronics Research Center, National Institute of Advanced Industrial Science and Technology, Central 5, 1-1-1 Higashi, Tsukuba, Ibaraki 305-8565, Japan; sei-uemura@aist.go.jp (S.U.); t-kamata@aist.go.jp (T.K.)

[3] Department of Image & Materials science, Graduate School of Advanced Integration Science, Chiba University, 1-33 Yayoi-cho, Inage-ku, Chiba 263-8522, Japan; nakamura.kazuki@faculty.chiba-u.jp

[4] School of Materials Science and Engineering, the Key Laboratory of Material Processing and Mold of Ministry of Education, Henan Key Laboratory of Advanced Nylon Materials and Application, Zhengzhou University, Zhengzhou 450001, China; dcs@gs.zzu.edu.cn (C.D.); liuxy@zzu.edu.cn (X.L.)

* Correspondence: bjldllj@hotmail.com (L.L.); Koban@faculty. chiba-u.jp (N.K.)

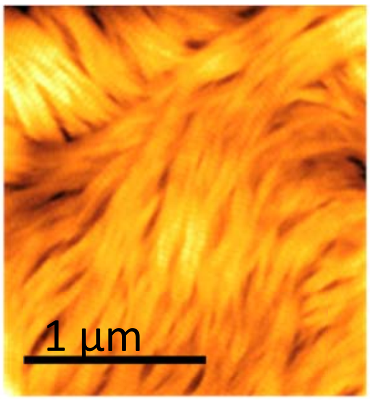

Fig. S1 AFM images of poly (γ-methyl-L-glutamate) (degree of polymerization: 440).

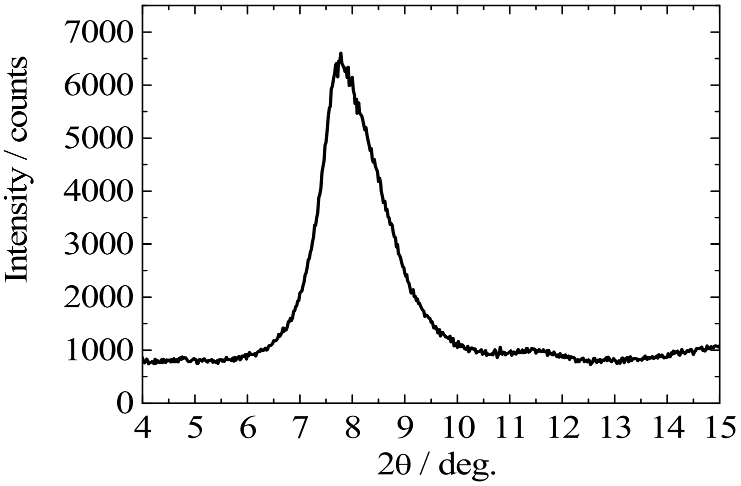

Fig. S2 XRD spectra of PMLG film (degree of polymerization: 440).

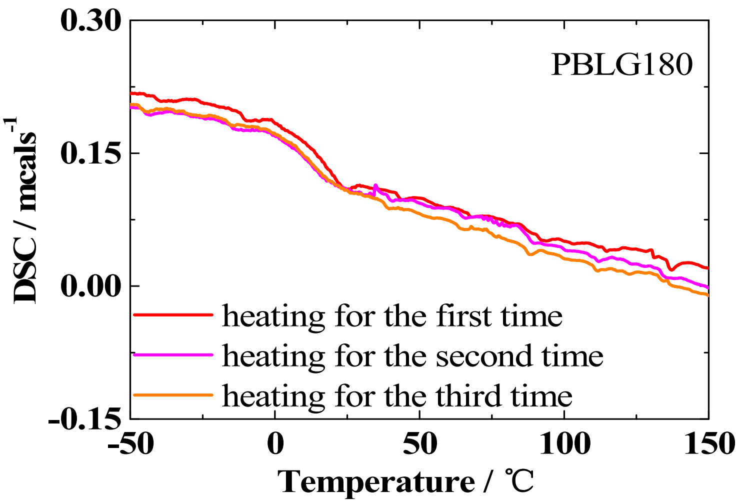

Fig.S3 DSC spectra of PMLG and PBLG.

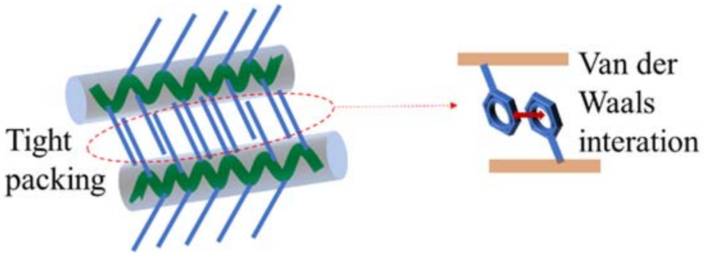

Fig. S4 Strong interaction causes high crystallinity in PBLG.

Table S1 FWHM and lattice spacing from XRD curves.

	FWHM	Lattice spacing
1:0	0.20	
8:2	0.30	12.3
6:4	0.48	
4:6	Amorphous	
